# Synthesis and Characterization of Porous MnCo_2_O_4_ for Electrochemical Determination of Cadmium ions in Water Samples

**DOI:** 10.1038/s41598-017-00748-x

**Published:** 2017-04-05

**Authors:** Murugan Velmurugan, Shen-Ming Chen

**Affiliations:** National Taipei University of Technology, Taipei, 106 Taiwan Republic of China

## Abstract

To utilize the maximum activity of nanomaterials, it was specifically synthesized by appropriate physicochemical properties. In that aspect, we have described the synthesis of porous MnCo_2_O_4_ by simple chemical route and applied for the selective detection of cadmium (Cd (II)). The as-prepared porous MnCo_2_O_4_ was characterized by scanning electron microscopy (SEM), transmission electron microscopy (TEM), Brunauer–Emmett–Teller (BET) adsorption isotherm, X-ray diffraction pattern analysis (XRD), Fourier transform infra-red spectroscopy (FT-IR), energy dispersive X-ray (EDX) and electrochemical techniques. The porous MnCo_2_O_4_ exhibited an excellent electrochemical behaviour and good analytical response towards the determination of Cd (II). Those analytical factors such as pH, deposition potential and deposition time are optimized by using differential pulse anodic stripping voltammetry (DPASV). A wide linear concentration range from 2.3 to 120 µg L^−1^, limit of detection (LOD) of 0.72 µg L^−1^ and the limit of quantification (LOQ) of 0.91 µg L^−1^ were achieved for determination of Cd (II). The selectivity of the developed sensor was explored in the presence of co-interfering ions. Also our sensor exhibits a good stability, reproducibility and repeatability. In addition, the practicability of proposed sensor was evaluated for the detection of Cd (II) in real water samples.

## Introduction

Discharge of toxic heavy metal ions to the environment causes most harmful issues to the human and ecosystem. For occasion, the industry of ceramic, semiconductors, pharmaceutical, metallurgical, agricultural and petrochemicals contaminates the surrounding water and soil^1–3^. The Cd is one of the toxic heavy metal, which causes nephrotoxicity, flu-like symptoms, renal tubular dysfunction, bone demineralization and cancers even for the intake of trace level through contaminated food and water^4–8^. Therefore, the international agency of research of cancer (IARC) classified the cadmium as a carcinogenic substance^9^. Moreover, the world health organization (WHO) and United States Environmental Production Agency (EPA) have defined the maximum level of Cd in drinking water as 0.003–0.005 µg L^−1^ ^10, 11^. Therefore, the detection of Cd is essential in water treatment and soil analysis. There are several methods such as inductively coupled plasma mass spectrometry^12, 13^, inductively coupled plasma atomic emission spectrometry^14^, atomic absorption spectrometry^15^, atomic fluorescence spectrometry^16^ and UV–Visible spectroscopy^17^. All the aforementioned methods offer good precision and high resolution, however, they are more expensive and needs trained technicians to handle the instruments. Moreover, the determination of Cd by those methods is time-consuming process. In contrast, the electrochemical methods have been accepted for the detection of heavy metal ions because of their excellent sensitivity, rapid analysis and low cost^18, 19^. Hence, the electrochemical method has emerged as a powerful technique for the detection of heavy metal ions when compared to other methods.

**Figure 1:**
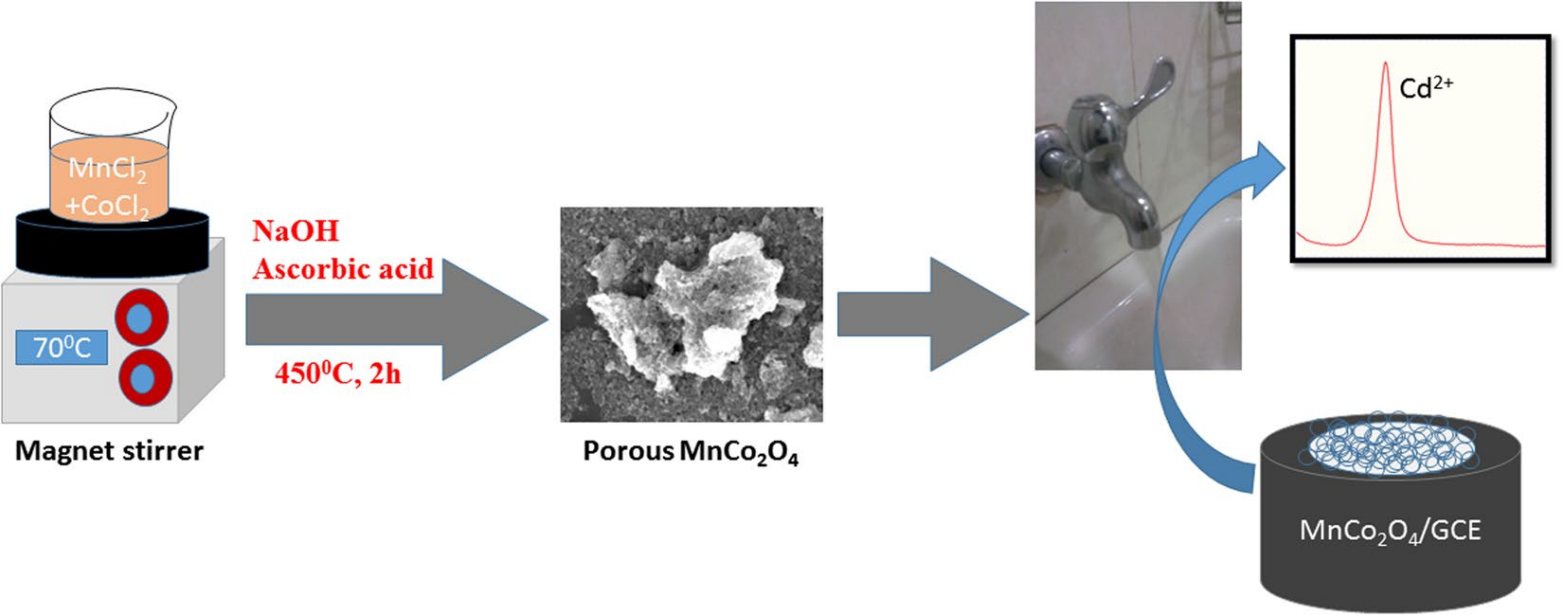
Preparation of porous MnCo_2_O_4_ and electrochemical detection of Cd^2+^.

In recent years, variety of porous metal oxides such as MgO^20^, Co_3_O_4_^21^ and NiO^22^ were reported for the electrochemical sensors. Because, these porous metal oxides have high surface area and open porous structure which helps in the detection of heavy metal ions. The transition metal oxides with a spinel structure have attracted more interest in the wide area of research field due to their unique properties such as magnetic, electrical and optical properties^23–25^. The common chemical formula of the spinel is AB_2_O_4_, where A and B are the divalent and trivalent metal ions, coordinated in tetrahedral and octahedral sites, respectively^26^. Among the materials developed for the Cd detection, the spinel metal oxides are the promising materials due to high earth abundance, low cost and environmentally friendly^27–29^. Hence, MnCo_2_O_4_ has been accepted as an alternative electrode material for the Cd sensor due to its excellent conductivity and tunable structural features^30, 31^. MnCo_2_O_4_ lies on the normal spinel family that consists of Mn^2+^ ions in tetrahedral sites, Co^3+^ ions in the octahedral sites and O^−2^ ions tend to coordinate both positions to frame the face centred cubic structure^32^. Besides sensors, MnCo_2_O_4_ has been widely utilized in alkaline fuel cells^33^, solid oxide fuel cells^34^, water treatment^35^ and glucose sensors^36^. In this work, we have demonstrated the synthesis of porous MnCo_2_O_4_ by simple chemical route at the low processing temperature. To the best of our knowledge, this is the first time we have reported the determination of Cd by porous MnCo_2_O_4._ Moreover, MnCo_2_O_4_ exhibited high sensitivity, excellent selectivity, wide linear concentration range and acceptable storage stability for the determination of Cd (II). The overall preparation and electrochemical pathway for the determination of Cd (II) was illustrated in Fig. 1.

## Results and Discussion

### Material characterizations

**Figure 2:**
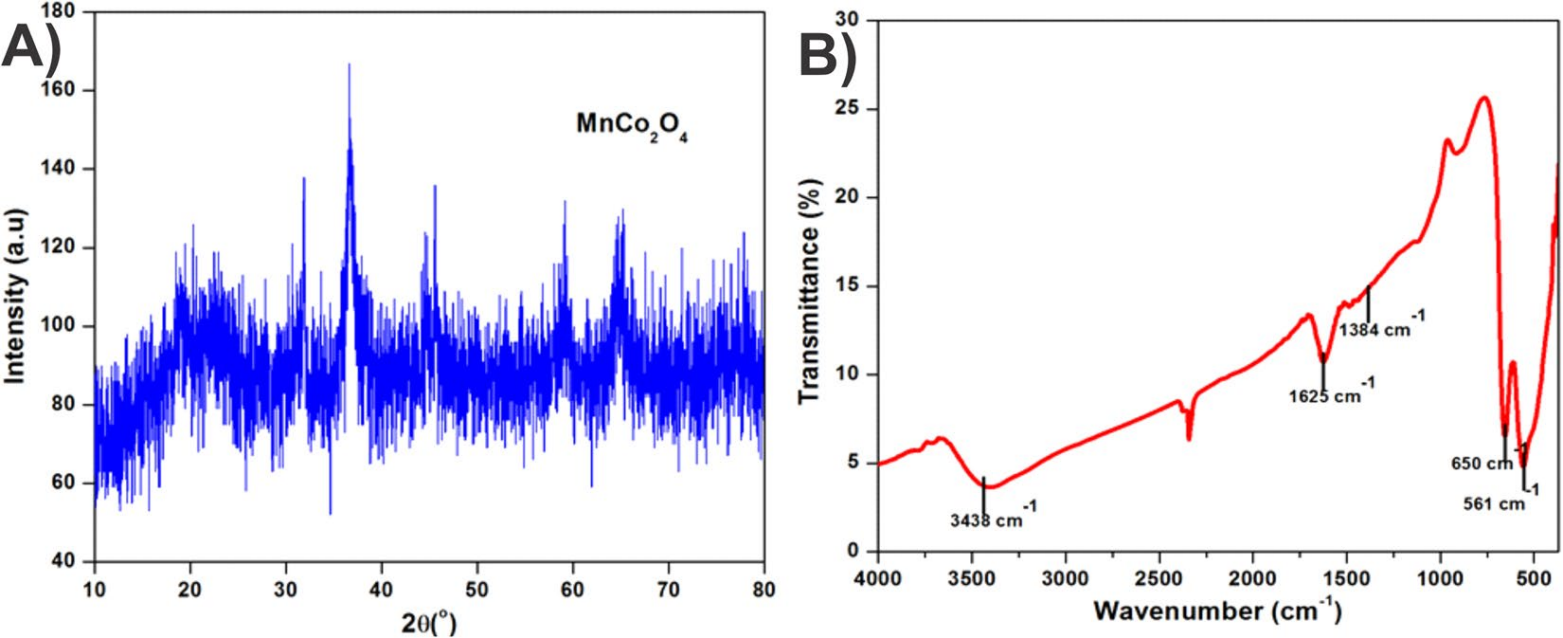
(**A**) XRD pattern (**B**) FTIR spectrum of porous MnCo_2_O_4_ spinel compound.

The crystallinity of as-prepared MnCo_2_O_4_ was confirmed by X-ray diffraction (XRD) analysis. Figure 2A depicts the XRD patterns of MnCo_2_O_4_ exhibiting the noisiest diffraction peaks which can be assigned as spinel compound. Those noises are due to the low crystallite size of the as-prepared compound. MnCo_2_O_4_ has a very noisy reflection peaks which indexed as a face centred cubic structure of MnCo_2_O_4_ with the space group fd3m (JCPDS No. 23-1237). The manganese and cobalt ions are well dispersive over octahedral and tetrahedral interstices to form the mixed valence ternary oxides as spinel MnCo_2_O_4_ crystal. The Debye-Scherrer formula (1) was used to calculate the average crystallite size of the as-prepared compound which exhibited the average crystallite size as 10 nm^37^.

1 $${\rm{D}}=0.9{\rm{\lambda }}/({\rm{\beta }}\,\cos \,{\rm{\theta }})$$

λ is the X-Ray wavelength, β is the full width at the half maximum and θ is the diffraction angle.

**Figure 3:**
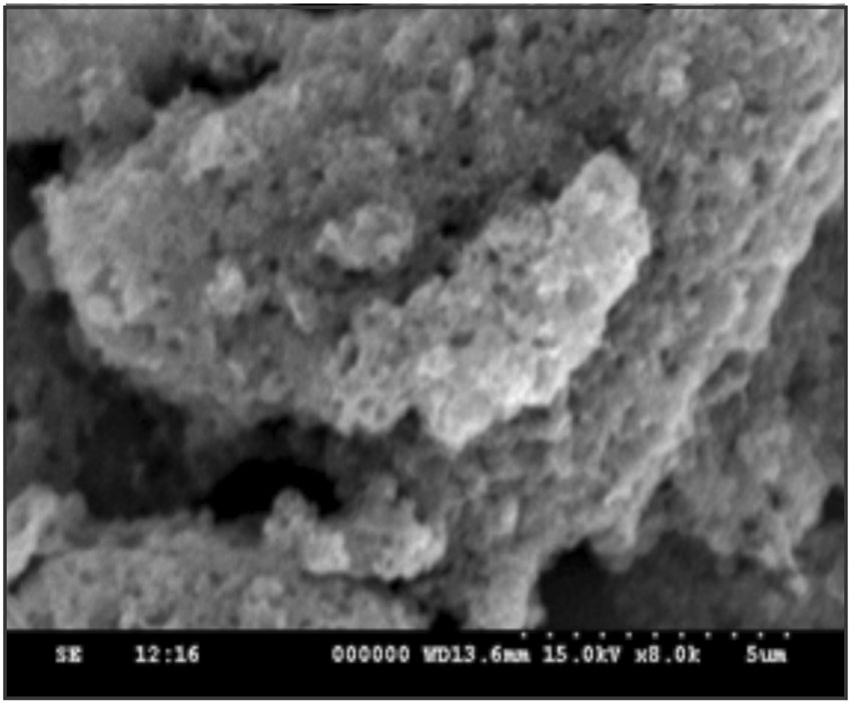
SEM image of porous MnCo_2_O_4_ spinel compound.

The FT-IR spectrum is a very essential tool to investigate the functional group analysis. Figure 2B displays the FT-IR spectrum of MnCo_2_O_4_, the stretching frequency at 3405 cm^−1^ reveals the broad band adsorption peaks for adsorbed water (H-O-H)^38^. It can be clearly seen that the angular deformation of the adsorbed water molecules band appeared at 1628 cm^−1^. The bands at 650 and 561 cm^−1^ are corresponding to the metal oxide characteristic peaks which revealed the formation of MnCo_2_O_4_ compound^39^. The surface morphology of MnCo_2_O_4_ was investigated by SEM. The SEM images of MnCo_2_O_4_ show the flake like morphology that consists of uniform macropores (Fig. 3). Such type of the surface morphology was having a good adsorption capacity towards toxic metal ions.

**Figure 4:**
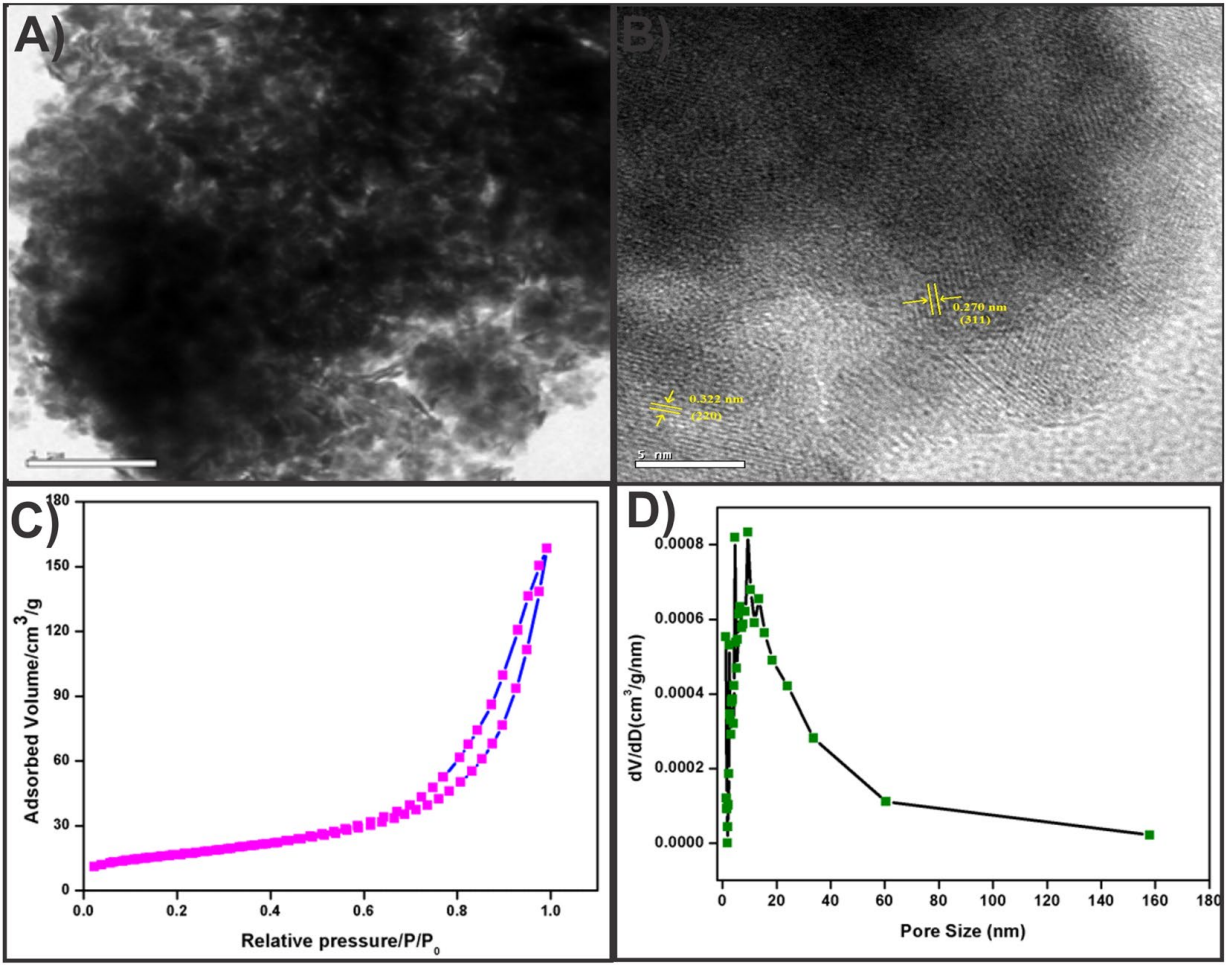
TEM image (**A**) and HR-TEM (**B**) of porous MnCo_2_O_4_ spinel compound (**C**) N_2_ adsorption–desorption isotherms and (**D**) the pore size distribution of the MnCo_2_O_4_ spinel compound.

The particle size and surface morphology of the porous MnCo_2_O_4_ were investigated by TEM. Figure 4A shows the TEM image of MnCo_2_O_4_ which exhibited the particles like porous morphology. The high magnification TEM image in Fig. 4B, displayed a distinct lattice fringes with an interplanar distance indexed to the crystal lattice (311) and (220) planes of spinel MnCo_2_O_4_. The specific surface area (SSA) and pore size of MnCo_2_O_4_ were examined by the N_2_ adsorption-desorption isotherms. MnCo_2_O_4_ adsorption isotherms and pore size distribution (PSD) curves were shown in Fig. 4(C,D). The specific surface area (58.82 m^2^ g^−1^) was calculated by Brunauer-Emmett-Teller (BET) method for the as-prepared MnCo_2_O_4_ compound. The wide pore size distribution range (2–33 nm) was observed and also the total pore volume was calculated as 0.2454 cm^3^ g^−1^ by using the BJH method.

**Figure 5:**
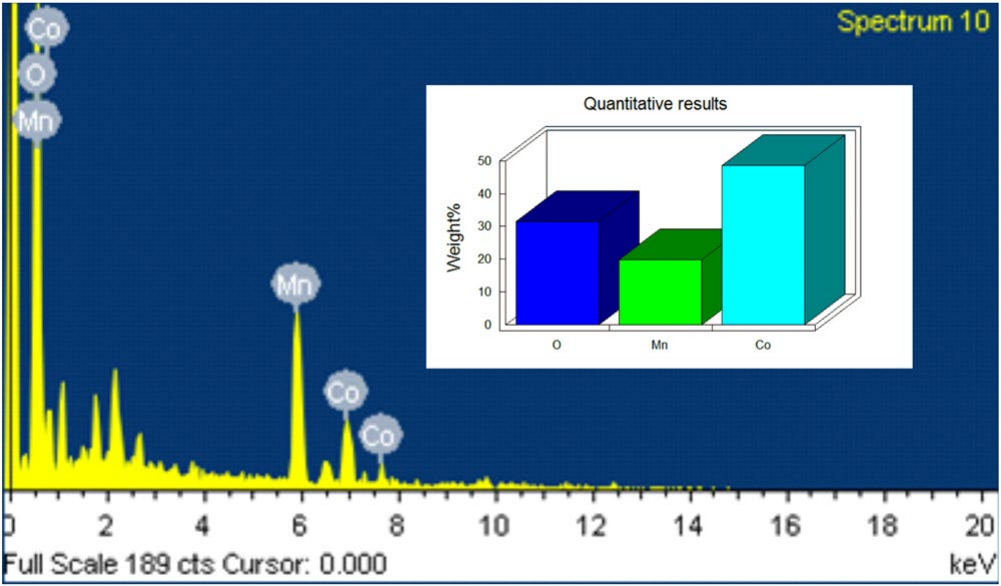
EDX spectrum and element weight % bar diagram of MnCo_2_O_4_.

Moreover, the presence of elements in MnCo_2_O_4_ compound was confirmed by EDX analysis and shown in Fig. 5. The EDX spectrum of MnCo_2_O_4_ exhibits the signal for Mn, Co and O with the weight percentage of 11.47%, 26.12% and 65.41%, respectively. These results confirmed that the as-prepared compound was porous MnCo_2_O_4_ spinel.

### Electrochemical behavior of Cd (II)

**Figure 6:**
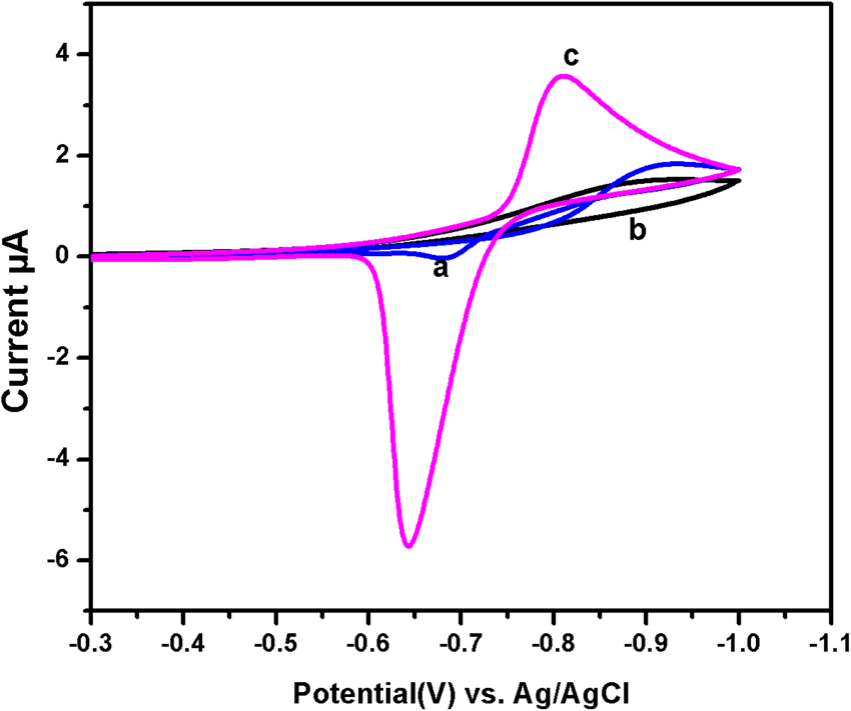
CV response of bare GCE in the presence of Cd (II) (a) MnCo_2_O_4_/GCE (c) and in the absence of MnCo_2_O_4_/GCE (b) in the acetate buffer (pH 5) solution containing 50 µg L^−1^ of Cd (II).

CV response of MnCo_2_O_4_/GCE was tested in the presence of 50 µg L^−1^ Cd (II) at a scan rate of 50 mVs^−1^ in acetate buffer solution. Figure 6 shows the electrochemical response of bare GCE (a) which exhibited the weak redox peak for 50 µg L^−1^ of Cd (II). It can be obviously seen that there were no peaks appeared for MnCo_2_O_4_/GCE (b) in the absence of Cd (II). However, a sharp and well-defined redox peak was observed for the porous MnCo_2_O_4_/GCE (c) with higher current response when compared with bare GCE. The obtained oxidation peak current from the porous MnCo_2_O_4_ was higher than that of the bare GCE. In addition, the porous MnCo_2_O_4_ provides a highly rough surface of the electrode which has a larger surface area with more active sites for Cd (II) accumulation.

### Optimization of parameters

**Figure 7:**
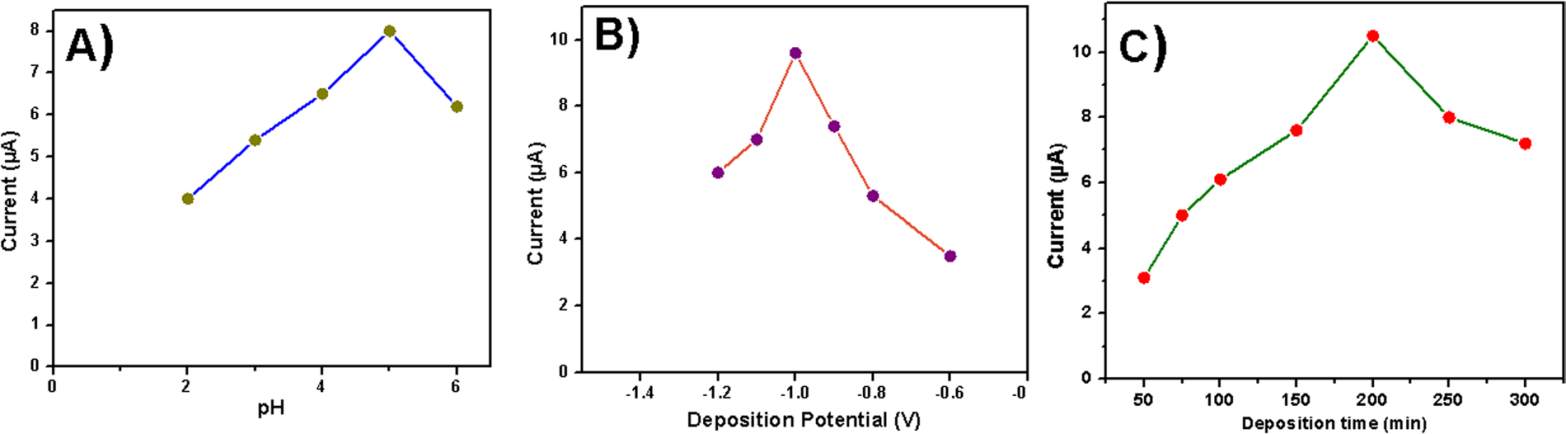
Effect of (**A**) pH of acetate buffer (**B**) Deposition potential of cadmium (**C**) Deposition time of cadmium on the DPSAV current in acetate buffer solution contained 50 µg L^−1^ of Cd (II) ion.

The effect of pH (acetate buffer solution) on the electrochemical performance of our proposed electrode was examined in the range of pH 2.0–6.0. It can be seen from the Fig. 7A, the maximum peak current was appeared at pH 5.0. Therefore, the pH 5.0 acetate buffer solution was used as electrolyte for the further detection of the Cd (II). The deposition potential is an important parameter in the detection of Cd (II). Therefore, the effect of deposition potential was investigated in presence of Cd (II) at pH 5.0 solution after 200 s of accumulation in the potential range from −0.6 to −1.2 V. Figure 7B depicts electrochemical activity for deposition potential showing the maximum current reached in the deposition potential of −1.0 V. Therefore, −1.0 V was fixed as the optimum deposition potential. The effect of the deposition time on MnCo_2_O_4_/GCE current response was also investigated from 50 s to 300 s. As shown in Fig. 7C, the current response was higher for the accumulation time of 200 s. Therefore, the 200 s of deposition time was chosen for the further experiment.

**Figure 8:**
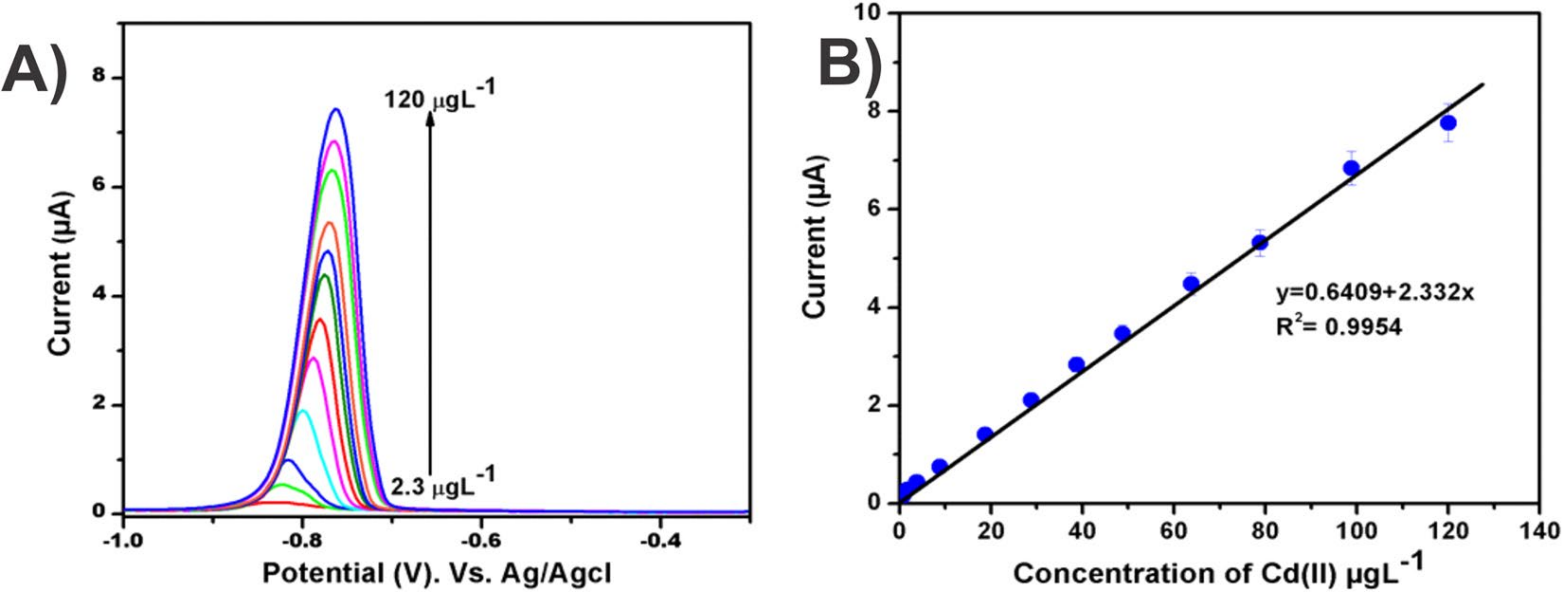
(**A**) Responses of MnCo_2_O_4_ modified GCE for the addition of different concentrations of Cd (II) from 2.3 to 120 µg L^−1^. (**B**) The corresponding linear plot for *I*_pa_ vs. concentration of Cd (II).

The determination of MnCo_2_O_4_ was tested by the successive additions of different concentrations of Cd (II) ions (Fig. 8A). Response of MnCo_2_O_4_/GCE was recorded towards various concentrations of Cd (II) from 2.3 to 120 µg L^−1^. The noticeable current response was appeared for the addition of 2.3 µg L^−1^ of Cd (II), further the peak current was increased with increasing the concentration of Cd (II). The gradual positive shift in the stripping potentials of Cd (II) might be due to the increase in the interfacial thickness of Cd (II)-MnCo_2_O_4_. A linear concentration range was obtained from 2.3 to 120 µg L^−1^ with the correlation coefficient of R^2^ = 0.9954 (Fig. 8B). The limit of detection was calculated as 0.72 µg L^−1^, limit of quantification was obtained as 0.91 µg L^−1^. The sensitivity of the proposed sensor was calculated by dividing the slope of the calibration plot by electroactive area, the calculated sensitivity is 7.7355 µA µg^−1 ^L cm^−2^. The analytical performances of the proposed sensor have been compared with previously reported other sensors (Table 1).

**Table 1:** Comparison for the analytical performance of other reported methods.

Modified electrode	Method	Linear range (µg L^−1^)	LOD (µg L^−1^)	Ref.
Nafion/Bi/NMC	DPASV	2–100	1.5	40
TM-MCM-41-CPE	Potentiometric	—	435	41
SnO_2_ quantum dots	CV	4990–44910	499	42
RGO/Bi	DPASV	20–120	2.8	43
G/PANI/PS-SPCE	SWASV	0–500	4.43	44
Bi/CNT	SWASV	2–100	0.7	45
2-benzothiazolethiol/amorphous silica- CPE	DPASV	—	11	46
Porous MnCo_2_O_4_/GCE	DPASV	2.3–120	0.72	Present work

NMC- Nitrogen doped microporous carbon, TM-MCM- Thiomorpholine-functionalized-Mobil Composition Matter, CPE- carbon paste electrode, RGO- Reduced graphene oxide, G/PANI/PS-Graphene/polyaniline/polystyrene nanoporous fibers, CV- Cyclic Voltammetry, DPASV-Differential pulse anodic stripping voltammetry, SWASV-Square-wave anodic stripping voltammetry.

### Interference study

**Figure 9:**
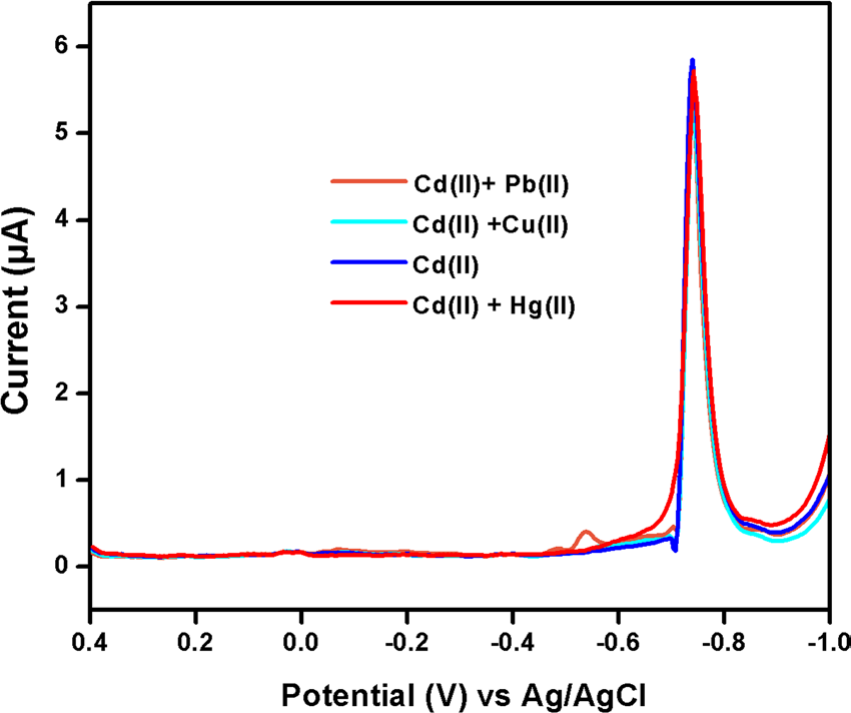
Effect of interference species on the detection of cadmium at MnCo_2_O_4_. Peak current response of 50 μg L^−1^ Cd (II) in the presence of 3-fold of Cu^2+^, Pb^2+^ and Hg^2+^.

In order to evaluate the selectivity of the fabricated sensor, it was investigated in the presence of various potentially interfering ions. Figure 9 shows the current responses for (50 µg L^−1^) Cd (II) in the presence of a 3-fold excess concentration of metal ions such as Cu^2+^, Pb^2+^ and Hg^2+^. The results showed that the interference signal was less than 1% for interfering ions. Therefore, the proposed sensor material selectively detected Cd (II) in the presence of interferents. These studies resulted that MnCo_2_O_4_ exhibited good selectivity for the detection of Cd (II) in the presence of other interference. Therefore, the proposed sensor material is suitable for the practical applications.

### Real sample analysis

In order to evaluate the reliability of proposed sensor, the determination of Cd (II) was examined in water samples. An optimized experimental condition was applied for the detection of Cd (II) in tap water. The standard addition method was used to detect Cd (II) and the calculated recovery results were presented in Table 2. It can be seen that the average recoveries of Cd (II) were 95.33–100.3% in tap water samples. These results evinced that the practicability of the proposed sensor towards the determination of Cd (II) in water samples.

**Table 2:** Determination of Cd (II) in water samples by MnCo_2_O_4_ modified GCE.

Sample	added (µM)	Found (µM)	Recovery (%)
1	3.0	2.85	95.00
2	15	15.02	100.13
3	50	49.95	99.90
4	100	98.89	98.89

### Stability, repeatability and reproducibility

The five different sensing electrodes were prepared under the same experimental conditions followed from section (Electrochemical behavior of Cd (II)) and observed the efficiency of the Cd (II) detection. The fabricated sensors showed almost same response for all the electrodes and revealed reproducibility with the RSD of 3.80% for the determination of Cd (II). These sensor electrodes were stored at room temperature over 30 days. After that it was retained about 90% of its initial response which confirmed that MnCo_2_O_4_ modified electrode has good storage stability. In addition, the sensor also has a good repeatability with RSD value of 3.2%, for the five repeated successive measurements of single modified electrode. These results disclose the proposed sensor material has excellent stability repeatability and reproducibility which endorsed that MnCo_2_O_4_/GCE is suitable for the practical applications.

## Conclusions

In summary, the honeycomb-like porous MnCo_2_O_4_ spinel was prepared from the simple facile method and subsequent calcination. The simple electrochemical technique was applied to detect the Cd ion by DPASV based on porous MnCo_2_O_4_ modified electrode. The developed sensor shows a wide linear concentration range from 2.3 to 120 µg L^−1^, lowest detection limit of 0.72 µg L^−1^ and excellent sensitivity of 7.7355 µA µg^−1^ L cm^−2^. The analytical performances of the developed sensor were comparable with the previous results. Moreover, MnCo_2_O_4_/GCE exhibits good storage stability, acceptable selectivity, excellent repeatability and reproducibility. In addition, the developed MnCo_2_O_4_/GCE validates the practicability towards the determination of Cd (II) in water samples.

## Experimental

### Chemicals and apparatus

Manganese chloride, cobalt chloride, cadmium nitrate, sodium hydroxide and ascorbic acid were obtained from Sigma–Aldrich. The supporting electrolyte of acetate buffer (pH 5) solution was prepared by using 0.05 M sodium acetate and glacial acetic acid. All the chemicals used were of analytical grade and used as received without purification. Cyclic voltammetry (CV) and differential pulse anodic stripping voltammetry (DPASV) measurements were performed by the CHI 900 electrochemical workstations. Scanning electron microscopy (SEM) was performed using Hitachi S-3000 H electron microscope, Fourier transform infrared spectroscopy (FT-IR) was carried out by using JASCO FT/IR-6600 instrument. The conventional three-electrode system was used for the electrochemical experiments, the modified glassy carbon electrode (GCE) was used as a working electrode (electrode area: 0.07 cm^2^), saturated Ag/AgCl used as a reference electrode and platinum electrode used as the auxiliary electrode.

### Synthesis of porous MnCo_2_O_4_ spinel oxide

For the preparation of porous MnCo_2_O_4_ composite, the molar ratio (1:2) of MnCl_2_ and CoCl_2_.6H_2_O were dissolved in 50 ml distilled water and stirred for 30 minutes. Then, 0.2 M NaOH was added drop-wise with constant stirring and after that 0.1 M ascorbic acid was slowly added to the suspension. Suspension temperature was maintained at 60–70° C for 1 hr. Finally, the precipitate was filtered and washed with ethanol and distilled water. The precipitate was dried at room temperature. After that the dried samples were calcined in a hot air oven at 450° C for 2 hr. Finally, the black color porous MnCo_2_O_4_ was obtained.

### Fabrication of electrode and operating condition

Glassy carbon electrode was precleaned with alumina powder and sonicated about 2 mins in ethanol and double distilled water. The adsorbed alumina slurry on the surface of GCE was removed by washing with double distilled water and dried in hot air oven. As-prepared composite was re-dispersed in water and sonicated to get well uniform suspension. A mirror cleaned GCE was fabricated with the suspension of MnCo_2_O_4_ by drop cast (6 µL) method and dried in a hot air oven at 35° C for 10 min. The fabricated MnCo_2_O_4_/GCE was further used for the electrochemical measurements.

All the experimental conditions were optimized by voltammograms. The electrochemical cell contains 10 mL of desired pH of acetate buffer (0.05 M) solution. The differential pulse Anodic stripping voltammograms were recorded from −0.3 to 1.1 V, applied potential of preconcentration of Cd (II) was −1.0 V and 200 s of preconcentration time. The calibration curve was obtained by plotting the peak current against deposition of cadmium potential range and deposition of cadmium accumulation time. The electrode was polished after each measurement with a clean paper.
